# Fertility-sparing uterine displacement for pelvic malignancies: surgical options and radiotherapy dosimetry on a human cadaver

**DOI:** 10.1186/s12957-024-03423-4

**Published:** 2024-06-03

**Authors:** Matteo Pavone, Laure Waeldin, Barbara Seeliger, Nicolò Bizzarri, Didier Mutter, Delphine Jarnet, Antonello Forgione, Noel Georges, Cherif Akladios, Giovanni Scambia, Jacques Marescaux, Lise Lecointre, Denis Querleu

**Affiliations:** 1 Institute of Image-Guided Surgery, IHU Strasbourg, 1 place de l’Hôpital, Strasbourg, 67091 France; 2 Research Institute against Digestive Cancer, IRCAD, Strasbourg, France; 3 UOC Ginecologia Oncologica, Dipartimento di Scienze per la salute della Donna e del Bambino e di Sanità Pubblica, Fondazione Policlinico Universitario A. Gemelli, IRCCS, Rome, Italy; 4 Radiation Therapy University Department, Institut de Cancérologie Strasbourg Europe (ICANS), 17 rue Albert Calmette, Strasbourg, 67200 France; 5 ICube UMR 7357-Laboratoire des Sciences de l’Ingénieur, de l’Informatique et de l’Imagerie, CNRS, University of Strasbourg, Strasbourg, 67000 France; 6 Department of Digestive and Endocrine Surgery, University Hospitals of Strasbourg, Strasbourg, 67000 France; 7 Medical Physics Unit, Institut de Cancérologie Strasbourg Europe (ICANS), 17 rue Albert Calmette, Strasbourg, 67200 France; 8 Department of Gynecologic Surgery, University Hospitals of Strasbourg, Strasbourg, France

## Abstract

**Background:**

Radio(chemo)therapy is often required in pelvic malignancies (cancer of the anus, rectum, cervix). Direct irradiation adversely affects ovarian and endometrial function, compromising the fertility of women. While ovarian transposition is an established method to move the ovaries away from the radiation field, surgical procedures to displace the uterus are investigational. This study demonstrates the surgical options for uterine displacement in relation to the radiation dose received.

**Methods:**

The uterine displacement techniques were carried out sequentially in a human female cadaver to demonstrate each procedure step by step and assess the uterine positions with dosimetric CT scans in a hybrid operating room. Two treatment plans (anal and rectal cancer) were simulated on each of the four dosimetric scans (1. anatomical position, 2. uterine suspension of the round ligaments to the abdominal wall 3. ventrofixation of the uterine fundus at the umbilical level, 4. uterine transposition). Treatments were planned on Eclipse® System (Varian Medical Systems®,USA) using Volumetric Modulated Arc Therapy. Data about maximum (Dmax) and mean (Dmean) radiation dose received and the volume receiving 14 Gy (V14Gy) were collected.

**Results:**

All procedures were completed without technical complications. In the rectal cancer simulation with delivery of 50 Gy to the tumor, Dmax, Dmean and V14Gy to the uterus were respectively 52,8 Gy, 34,3 Gy and 30,5cc (1), 31,8 Gy, 20,2 Gy and 22.0cc (2), 24,4 Gy, 6,8 Gy and 5,5cc (3), 1,8 Gy, 0,6 Gy and 0,0cc (4). For anal cancer, delivering 64 Gy to the tumor respectively 46,7 Gy, 34,8 Gy and 31,3cc (1), 34,3 Gy, 20,0 Gy and 21,5cc (2), 21,8 Gy, 5,9 Gy and 2,6cc (3), 1,4 Gy, 0,7 Gy and 0,0cc (4).

**Conclusions:**

The feasibility of several uterine displacement procedures was safely demonstrated. Increasing distance to the radiation field requires more complex surgical interventions to minimize radiation exposure. Surgical strategy needs to be tailored to the multidisciplinary treatment plan, and uterine transposition is the most technically complex with the least dose received.

## Synopsis

Uterine displacement procedures are currently experimental. As demonstrated for ovarian transposition, moving the uterus out of the pelvis prior to radiotherapy is a fertility-sparing option for young women with pelvic malignancies.

## Introduction

The final goal of cancer care is achieving a cure. While significant progress in oncology resulted in higher cure rates and prolonged survivorship, some aspects, such as sexual, ovarian, and reproductive functions in young women, have often been overlooked by physicians and patients alike [1]. Estimations indicate that there will be a 13,5% increase in new cases of pelvic malignancies (cancers of anus, rectum, vagina, cervix) among women under the age of 44 worldwide, particularly in low-income countries, from 2020 to 2040 [2, 3]. Concurrently, social changes are causing a rise in the average age of first pregnancies, making fertility-sparing approaches crucial, especially when pelvic malignancy requires chemo- and/or radiotherapy (RT) [4]. Pelvic irradiation can damage reproductive organs directly and impair ovarian and uterine function. The toxic radiation dose for ovaries is known and decreases with age [5] while there is less data on the dose causing permanent loss of uterine function. Some authors suggest a mean uterine radiation dose of 14 Gy compatible with pregnancy [6], while others advise to not exceed 20–25 Gy, in comparison with other organs [7]. Advanced radiotherapy techniques like intensity-modulated and image-guided radiotherapy enable precise targeting, but due to the nature of external beam treatment, a portion of the dose always reaches the uterus. Similar to ovarian transposition, a procedure to preserve ovarian function before pelvic irradiation [8, 9], some authors propose to displace the uterus to prevent radiation damage and preserve fertility [10]. Reported uterine displacement techniques include: uterine suspension (US), uterine ventrofixation (UV) and uterine transposition (UT) [10]. Although several uterine displacement procedures exist, there is no consensus on the optimal balance between the largest dose reduction and technical reproducibility. This study aims to assess the uterine displacement techniques and the respective received radiation doses at the level of the uterus on a female human cadaver.

## Methods

### Surgical procedure

The frozen female human cadaver of a body donor (Anatomical Institute, University Hospitals of Strasbourg) was warmed at ambient temperature for 12 h. Pre-operative magnetic resonance imaging (MRI) scan was performed to check the suitability of the body within the scope of the study, in particular the presence of a normal uterus. On the same model in lithotomy position, the following procedures were performed subsequently: 1. uterine suspension of the round ligaments to the abdominal wall, 2. uterine ventrofixation of the fundus at the level of the umbilical line, 3. uterine transposition. Before performing the uterine displacements, the right ovary was transposed in the upper abdomen to assess the radiation dose received compared with the contralateral organ in the anatomical position. A CT-scan was performed after each uterine displacement. The surgical procedures were performed at the IHU Strasbourg Institute of Image-Guided Surgery in a hybrid operating room equipped with a CT-scan able to evaluate the organs positions according to anatomical landmarks [11]. Metallic clips were applied to ovaries, and uterine arteries as reference for subsequent radiologic image interpretation. The surgical procedure was carried out by two expert gynaecologic oncologists (DQ, LL) and a gynaecologic oncology research fellow (MP), and assisted by a visceral surgeon (BS).

### Delineation and dosimetry planning

The four CT-scans were imported into the delineation software Somavision V17.4 (Eclipse® System, Varian Medical Systems ®, Palo Alto, CA, USA). General pelvic organs at risk (including bladder, rectum, bowel bag, femoral heads) and gynecological organs at risk (uterus, cervix and ovaries) were delineated in each of the CT-scans according to the contouring guidelines for radiation therapy [12]. Rectal Clinical Target Volume (CTV) and anal canal CTV were defined according to international recommendations [13, 14], with respective simulated doses of 50 Gy delivered to the rectal tumor and mesorectum and 45 Gy to the prophylactic lymph node volume (internal iliac areas, presacral area), as well as 64 Gy to the anal canal tumor and 45 Gy to the prophylactic lymph node volume (inguinal, external iliac, obturator, internal iliac, presacral and mesorectal areas). Planning Target Volume (PTV) was obtained by adding a 5 mm margin around the CTV. A soft tissue density was attributed to the air bubbles present in the cadaver. Treatments were planned on Eclipse® System V17.4 (Varian Medical Systems ®, Palo Alto, CA, USA) using Volumetric Modulated Arc Therapy and Accuros algorithm (Dose to medium in medium). A total of 8 treatment plans was calculated, one for each of the four uterine positions (anatomical, US, UV, UT) in the simulations of both rectal cancer and anal canal cancer. Target volume coverage was correct, and the constraints on organs at risk used in our department were respected. The maximum (Dmax) and mean (Dmean) doses, and the V14 (i.e., the volume of uterus receiving 14 Gy) were registered for the uterus in each of the 8 dosimetry plans, and Dmax and Dmean were registered cumulatively for both ovaries.

## Results

### Surgical procedures

**Fig. 1:**
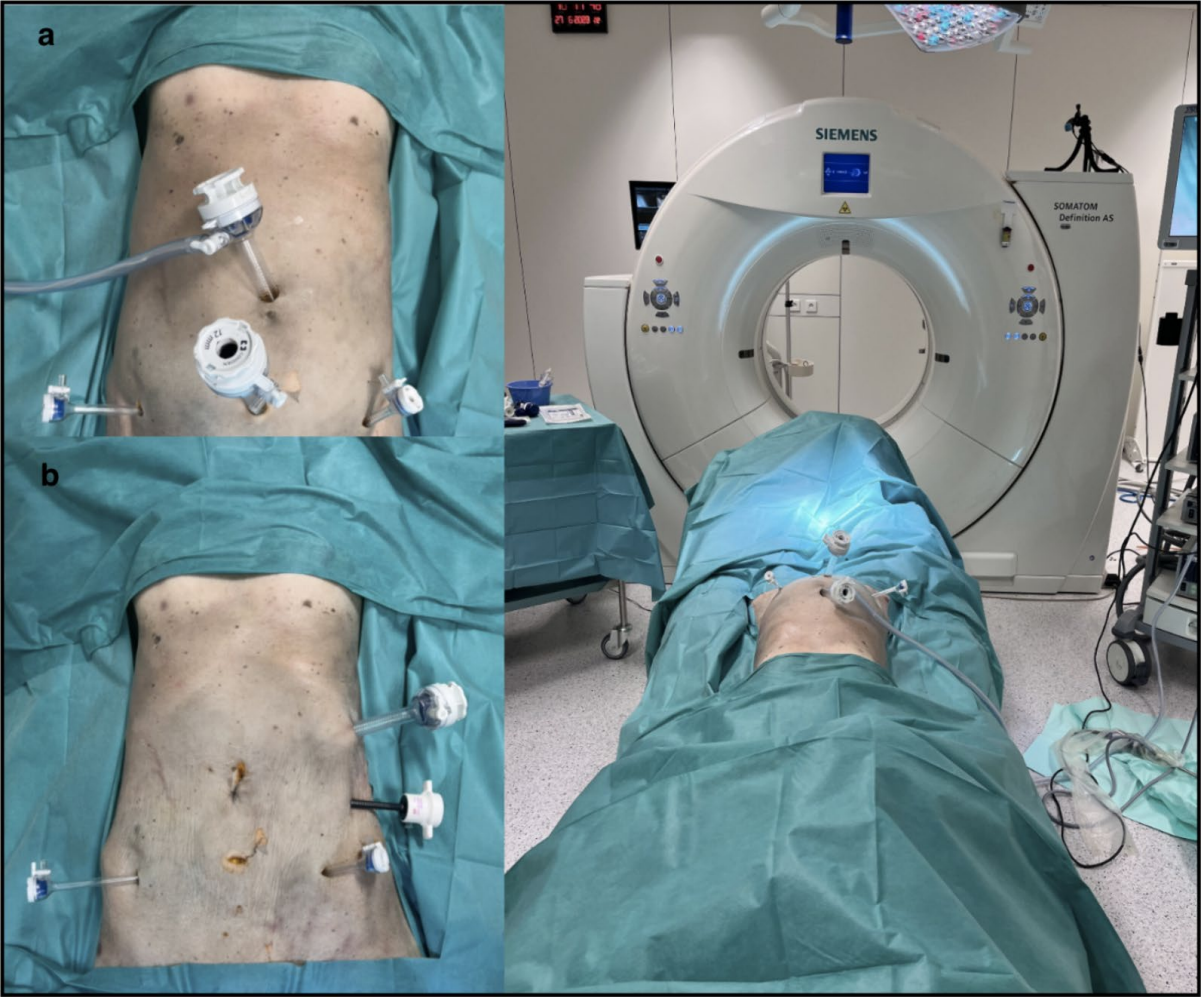
**a** trocar placement for US **b** trocar placement for UV and UT

The surgical approaches for uterine displacement were laparoscopically performed step by step and completed without complications on a human cadaveric model in lithotomy position. The right ovary was transposed according to the standardized technique proposed by Bizzarri et al. [9]. The first displacement procedure involved moving the uterus out of the pelvis either by suspension of the round ligaments or by ventrofixation of the uterine fundus at the level of the umbilicus. The second as described by Ribero et al. [15] consists of the uterine transposition with an -ostomy of the cervix to the umbilicus. After each individual procedure, a CT scan was performed for further dosimetry estimation. The US was performed with the trocar setting shown, a 12 mm port in the umbilicus for the laparoscope, two 5 mm trocars in the flanks and a 12 mm suprapubic trocar **(**Fig. 1a**)**. For UV and UT, the access was changed with trocars on the left flank (Fig. 1b). Suspension of the round ligaments was technically the easiest procedure. A straight needle with a T-lift system or a non-absorbable thread can be used to pass through the round ligaments bilaterally and through the uterine fundus to attach it to the anterior abdominal wall. After the end of the radiotherapy treatment the uterus is usually repositioned in the anatomical position. For the ventrofixation at the level of the umbilicus, uterine mobilization was achieved by incising the posterior peritoneum of the Douglas Pouch, allowing for the fundus to be moved up to the level of the umbilicus while preserving the vascular supply, which is possible due to the natural elasticity of the vagina. Uterine transposition as proposed by Ribeiro et al. is a more complex surgical procedure. The anterior leaf of the broad ligament was opened bilaterally until its junction with the uterus, the round ligaments were transected, and the posterior peritoneum dissected. The vesico-uterine space was opened, and the bladder reflected. The uterine vessels were clipped and cut in their corporal portion along their junction to the cervix. After insertion of a vaginal sponge, colpotomy was performed and the vaginal cuff closed with separate stitches of absorbable sutures. Care was taken to avoid grasping the Fallopian tubes, the infundibulo-pelvic ligaments (IP) or utero-ovarian ligaments to avoid damage to the uterine vascular supply or the tubes which can impair future fertility. The uterus was then transposed to the upper abdomen. Once the capnoperitoneum was deflated, the umbilical trocar incision was enlarged and the cervix anastomosed to the fascia by means of six separate polypropylene 3 − 0 sutures. The uterus is then repositioned in the pelvis and the cervix re-sutured to the vagina after the end of the radiotherapy treatment.

### Dosimetric simulation

**Fig. 2:**
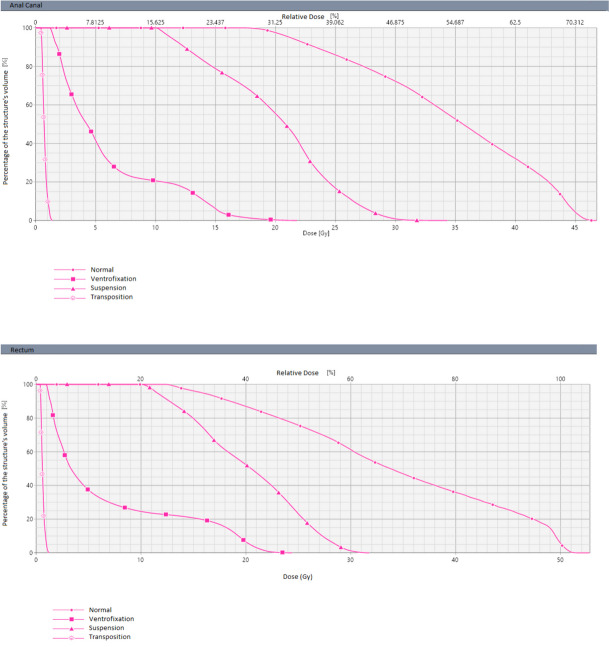
Dose-Volume uterine histograms

For the rectal cancer scenario 50 Gy to the tumor and 45 Gy to the lymph node drainage areas were delivered while for the anal cancer scenario 64 Gy to the tumor and 45 Gy to the lymph node drainage areas. Tables 1, 2 and 3 report dosimetry data (Dmax, Dmean and V14Gy) for uterus and ovaries for each procedure and both scenarios (anal and rectal cancer) in detail. Dose-Volume histograms of the uterus are shown in Fig. 2.

**Table 1:** Dmax, Dmean and V14Gy received by the uterus according to the position in the abdomen for anal and rectal cancer dose simulation

Uterine Position	Anus	Rectum	Graphical representation
**Anatomical uterine position**			
* Dmax*	46,7 Gy	52,8 Gy	
* Dmean*	34,8 Gy	34,3 Gy	
* V14Gy*	31,3 cc	30,5 cc	
**Round ligament suspension to the abdominal wall**			
* Dmax*	34,3 Gy	31,8 Gy	
* Dmean*	20.04 Gy	20,2 Gy	
* V14Gy*	21,5 cc	22.0 cc	
**Fundus ventrofixation at the umbilical level**			
* Dmax*	21,8 Gy	24,4 Gy	
* Dmean*	5,9 Gy	6,8 Gy	
* V14Gy*	2,6 cc	5.5 cc	
**Uterine transposition**			
* Dmax*	1,4 Gy	1,8 Gy	
* Dmean*	0,7 Gy	0,6 Gy	
* V14Gy*	0.0 cc	0.0 cc

**Table 2:** Rectal cancer dose simulation. Dmax and Dmean received by the ovaries. R: right (transposed); L: left (not transposed); L + R: cumulative dose for left and right ovaries

Rectal Cancer			
Uterine Position	R Ovary	L Ovary	L + R Ovary
**Anatomical uterine position**			
* Dmax*	36.9 Gy	23.72 Gy	36.9 Gy
* Dmean*	17 Gy	11.13 Gy	14.9 Gy
**Round ligament suspension to the abdominal wall**			
* Dmax*	20.3 Gy	14 Gy	20.3 Gy
* Dmean*	10.3 Gy	9.7 Gy	9.9 Gy
**Fundus ventrofixation at the umbilical level**			
* Dmax*	10.5 Gy	26 Gy	26.7 Gy
* Dmean*	6.1 Gy	8.1 Gy	7.7 Gy
**Uterine transposition**			
* Dmax*	9 Gy	14.4 Gy	14.4 Gy
* Dmean*	5.3 Gy	6.1 Gy	5.8 Gy

**Table 3:** Anal cancer dose simulation. Dmax and Dmean received by the ovaries. R: right (transposed); L: left (not transposed); L + R: cumulative dose for left and right ovaries

Anal Cancer			
Uterine Position	R Ovary	L Ovary	L + R Ovary
**Anatomical uterine position**			
* Dmax*	42.5 Gy	40 Gy	42.5 Gy
* Dmean*	36.9 Gy	33 Gy	35 Gy
**Round ligament suspension to the abdominal wall**			
* Dmax*	43 Gy	45,3 Gy	45.3 Gy
* Dmean*	28 Gy	14.5 Gy	18.9 Gy
**Fundus ventrofixation at the umbilical level**			
* Dmax*	13.1 Gy	46.4 Gy	46.4 Gy
* Dmean*	7.2 Gy	16 Gy	14.1 Gy
**Uterine transposition**			
* Dmax*	9.6 Gy	15 Gy	15 Gy
* Dmean*	5.6 Gy	7.9 Gy	7.1 Gy

## Discussion

The dose simulations specific to each of the uterine displacement techniques revealed that uterine transposition provided the most protection for irradiation in both scenarios (rectal and anal cancer), with a Dmax of only 1,8 Gy and 1,4 Gy, respectively. For uterine ventrofixation, none of the simulations exceeded a mean dose of 14 Gy, and a maximum dose of 25 Gy. Given that this approach is technically simpler than uterine transposition, possible complications, side effects and the need for a secondary surgery can be minimized as the ventrofixation can be reversed during the colorectal surgical procedure after completion of neoadjuvant treatment. The dose simulations for the technically simplest procedure of uterine suspension revealed Dmax and Dmean potentially compromising the uterine function in both scenarios (Anus 34,3 Gy and 20.04 Gy; Rectum 31,8 Gy and 20,2 Gy). Certain limitations were encountered during dose calculations. The presence of air in the cadaver posed challenges to dosimetry, and organ contours were approximated. Moreover, organs exhibited a displacement from normal anatomy, possibly attributable to thawing, while the vessels remained in situ.

The present study goes into detail by implementation of all known techniques and comparing their feasibility and received radiotherapeutic doses within a human cadaveric model, and is based on a prior systematic review and investigation involving dose calculations on a simulation of uterine position on actual treatment plans for two patients with rectal and anal cancer [10], with coherent results. Uterine transposition is a complex procedure, with a longer operative time, increased blood loss, and a risk of complications similar to those of a standard hysterectomy (vascular and ureteral injuries, intestinal or bladder lesions, etc.), as well as those related to organ devascularization (uterine necrosis or cervical stenosis) revealed by a recent systematic review [10]. In the last decade, fertility-sparing treatments gained increasing attention. With the advancement in oncological care and increased rate of cancer survivorship, the global welfare of patients is now considered pivotal. To date, the reproductive function is crucial for young women with an oncological diagnosis and conception desire. Advancement in treatment can now be curative for patients but irradiation can permanently damage the uterine and ovarian function [9]. Although the precise dose-effect association for uterine reproductive impairment remains unknown, evidence suggested that a dose of 14 Gy is compatible with gestation. Some experts recommended limiting the received dose to 20–25 Gy, drawing parallels with other glandular organs such as the parotid gland [16]. For more than a decade, several authors reported uterine displacement techniques as potential procedures to preserve uterine function in patients undergoing radiotherapy for pelvic malignancies [10]. Yet, these methods are not commonly employed in clinical practice. In 2010, Querleu et al. [17] were the first to describe the uterine suspension and ventrofixation in rectal cancer patients in a laparoscopic approach and suggested the dissection of posterior compartment peritoneum when the vaginal elasticity was insufficient to move the uterus out of the pelvis otherwise. Since then, only few case reports were published reporting encouraging results and absence of major procedure-related complications [18, 19]. In one case, a positive obstetric outcome was described after ventrofixation and ovarian transposition before radiochemotherapy with 45 Gy for rectal cancer. In 2017, uterine transposition was proposed as a displacement technique [20]. In this case the cervix, detached from the vagina as for trachelectomy, can be knotted to the lateral flank when menses are suppressed hormonally, or sutured to the umbilicus with menstrual bleeding through the umbilical scar. In the prospective study, eight patients (seven with rectal cancer and one with pelvic liposarcoma) underwent UT with the uterus successfully preserved and repositioned after radiotherapy treatment in six patients [15]. Two out of the three patients who attempted to conceive succeeded spontaneously, delivering healthy babies by cesarean section. However, cervical necrosis was reported as a post-surgical complication in 37.5% of patients. UT was also reported to be performed robotically and in pre-puberal patients [21, 22]. Although this technique was mainly indicated for digestive cancers, uterine transposition after trachelectomies was reported off-label for cervical cancer and in view of a strong patient desire [21, 23, 24]. Ovarian transposition is a mandatory surgical step before US and UV while it is implicitly performed in the UT technique with uterus and adnexa moved as a whole into the upper abdomen. Our dose simulations showed that transposing ovaries is essential in UV (rectal cancer: transposed ovary Dmax 10,5 Gy vs. 26 Gy in anatomical position; anal cancer: transposed ovary Dmax 13,1 Gy vs. 46,4 Gy) while there were no differences in the US. The latter might be due to the indirect stress on the infundibulo-pelvic ligaments which brings the ovaries in closer proximity to the pelvis. Due to the common requirement for radiotherapy in pelvic malignancies and the associated worries about radiation-induced damage to the reproductive organs in young women with pelvic malignancies who aim for fertility, the findings of this study offer valuable insights into selecting the most suitable surgical approach before pelvic irradiation. It is worth highlighting that instances of successful pregnancies after radiotherapy for rectal cancer with well-defined doses are infrequently reported. In our investigation, we calculated the doses received by the uterus for each performed technique. Notably, our results consistently demonstrated that the average dose for UV and UT remained below 14 Gy, irrespective of whether the dosimetry planning was for rectal or anal canal cancer.

In the literature uterine transposition is the only approach with reported major intra- and postoperative complications, with no cases for US/UV. Additionally, only three cases of delivery are described after these displacement techniques. The limited data does not provide definitive conclusions on which technique is generally preferable.

## Conclusion

Based on the present study, both uterine ventrofixation and uterine transposition are practicable techniques, demonstrating compatibility with simulated radiation doses favourable to pregnancy. The exploration of uterine displacement methods as a strategy to preserve fertility in young patients diagnosed with rectal or anal cancer warrants further investigation. However, the available obstetric results lack the robustness needed to draw definitive conclusions. To address this gap, a prospective clinical trial with a significative cohort size to assess the efficacy of uterine displacement in enhancing fertility outcomes is imperative.

## Data Availability

No datasets were generated or analysed during the current study.
